# 573. Digital Patient/Caregiver Education: A Tool to Improve COVID-19 Vaccination Rates and Confidence Among the Public

**DOI:** 10.1093/ofid/ofab466.771

**Published:** 2021-12-04

**Authors:** Amy Larkin, Allison Armagan

**Affiliations:** 1 Medscape Education, Nicholasville, Kentucky; 2 Medscape, New York, New York

## Abstract

**Background:**

Due to patient hesitancy surrounding the COVID-19 vaccination initiative, the public needs accurate and timely education that encourages partnership with medical professionals.

**Methods:**

This study assessed the impact of online patient/caregiver education on knowledge, confidence and intent to act. The educational intervention consisted of 4 activities published on a dedicated COVID-19 learning center on WebMD Education portal from April-May, 2021. The activities were comprised of text and integrated visuals, with 3 of the activities being further customized with a patient or healthcare professional (HCP) video commentary. Demographic questions were asked prior to each activity. Knowledge questions were asked both before and after to assess learning gains. Intent to change and confidence questions were asked at the end of each activity. Absolute improvements were calculated for pre/post questions. An initial data pull was conducted on 6/7/2021 for the purpose of this abstract, and data for the complete analysis will be collected until approximately 8/7/21.

**Results:**

To date, 14,911 learners (3,579) of which responded to the pre/post questions) have participated in the activities, and have demonstrated improvements in knowledge and high levels of confidence and intent to act (Figure). Activity 1: COVID-19 Vaccines: Covering the Basics. Demographics (n=155): 50% male; 41% White, non-Hispanic, 30% Asian; 52% over the age of 54. Activity 2: Understanding the Why, Who, and When of COVID-19 Vaccines. Demographics (n=2,325): 66% female; 51% White, non-Hispanic, 18% Asian; 54% over the age of 54. Activity 3: What to Expect When You Get the COVID-19 Vaccine. Demographics (n=500): 66% female; 49% White, non-Hispanic, 22% Asian; 56% over the age of 54. Activity 4: What Have You Heard about Herd Immunity and COVID-19. Demographics (n=599): 63% female; 53% White, non-Hispanic, 25% Asian; 53% over the age of 54.

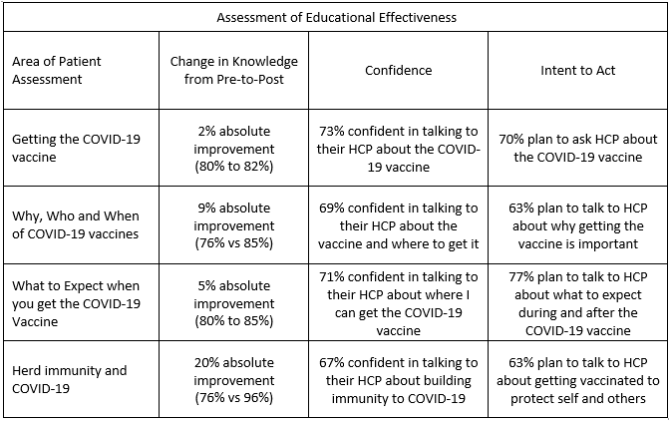

**Conclusion:**

The metrics and outcomes gathered in this assessment are a strong indicator that online patient/caregiver activities on WebMD Education improved knowledge and confidence and prompted intent to act related to COVID-19 vaccines. These findings highlight the potential for well-designed online education to overcome vaccine related challenges of the COVID-19 pandemic.

**Disclosures:**

**All Authors**: No reported disclosures

